# Editorial: Exploring the neural mechanisms of sensory-cognitive associations: bridging sensory perception and higher cognitive functions

**DOI:** 10.3389/fnins.2025.1698402

**Published:** 2025-09-29

**Authors:** Hongyu Qu, Zhilin Zhang

**Affiliations:** ^1^School of Psychology, Shenzhen University, Shenzhen, China; ^2^Research Center for Medical Artificial Intelligence, Shenzhen Institutes of Advanced Technology, Chinese Academy of Sciences, Shenzhen, China

**Keywords:** sensory-cognitive integration, multi-modal, cognitive-affective regulation, computational modeling, neuroimaging

## Introduction

The dynamic interplay between sensation and cognition lies at the core of human experience. For centuries, philosophers and scientists have sought to understand how raw sensory signals are transformed into meaningful perceptions, which in turn shape thoughts, emotions, and behaviors. Despite major advances, the neural mechanisms linking sensation and cognition remain a key challenge in modern neuroscience.

This Research Topic was designed to address this gap, bringing together studies that illuminate the neural choreography of sensory-cognitive integration from multiple perspectives. Interdisciplinary contributions address the neural and cognitive mechanisms underlying human perception, cognition, and behavior. By integrating evidence from neuroimaging, electrophysiology, computational modeling, and psychophysiological approaches, the Research Topic highlights advances bridging fundamental neuroscience with clinical and applied perspectives. Collectively, these studies argue that cognition is not merely a passive recipient of sensory input but is embedded within, and actively shapes, sensory processing from its earliest stages.

## Sensory processing and multisensory integration

A central question concerns how sensory features are processed and integrated into coherent perceptions. Wang et al. demonstrated hemispheric specialization in extracting visual symmetry under noisy conditions, reflecting sophisticated neural mechanisms for pattern recognition. Their findings reveal distinct neural components associated with different stages of visual perception, contributing to the understanding of how the brain processes symmetry. Wu et al. showed that spatial frequency dynamically modulates early audiovisual integration, highlighting rapid and reciprocal sensory interactions.

Attention and salience further determine which sensory signals are prioritized. Fujita et al. developed a computational framework linking visual salience to eye-movement behavior. Their model effectively predicts eye-movement patterns and provides insights into how neuronal abnormalities can influence visual salience, bridging computational neuroscience with behavioral data. Zhang et al. revealed that tactile imagery engages overlapping parietal networks with actual touch but exhibits distinct temporal dynamics, illustrating that tactile perception and imagery involve separate neural pathways despite both relying on somatosensory processing.

## Cognitive-affective regulation and large-scale networks

The sensory-cognitive dialogue extends into self-perception and emotional regulation. Aki et al. conducted resting-state functional magnetic resonance imaging analyses to investigate the neural correlates of self-esteem. They identified robust functional connectivity between the left dorsolateral prefrontal cortex and posterior cerebellum, as well as associations with other brain regions involved in social cognition and emotional regulation, providing insights into the neural basis of self-esteem. Shibata et al. examined the functional connectivity of key brain networks in individuals with non-problematic internet use. They found alterations in the default mode, salience, and frontoparietal networks, which were associated with mood regulation, highlighting the impact of internet usage patterns on cognitive and emotional processing.

## Clinical perspectives and methodological advances

Top–down modulation of sensory processing is particularly prominent in clinical and translational contexts. Golshan and Mickleborough reviewed neurophysiological mechanisms through which meditation influences brain activity in response to acute and chronic pain. Their review categorized general models explaining how meditation alters cortical responses to painful stimuli and identified key brain regions consistently implicated in pain modulation, providing a framework for understanding the neural effects of meditation on pain processing. Nicolardi et al. investigated pain perception in children and adolescents with autism using quantitative sensory testing (QST) protocols. They measured central reactivity to painful stimuli via electroencephalographic (EEG) recordings, revealing altered sensory reactivity and elucidating mechanisms of neurodivergent sensory-cognitive integration in this population.

Technological advances further enhance research capabilities. Chen et al. developed an advanced gustometer for reliably recording gustatory event-related potentials in healthy young adults. Their study aimed to improve understanding of gustatory processing and its neural correlates, providing methodological innovations that support research on sensory perception and its neural mechanisms.

## Conclusion

Collectively, these studies provide a comprehensive overview of the neural mechanisms that bridge sensory perception and higher cognitive functions. They highlight the complexity and specialization of neural pathways involved in processing sensory inputs and integrating them into cognitive and emotional responses. The diverse methodologies employed, ranging from electrophysiological recordings to neuroimaging and computational modeling, underscore the interdisciplinary approaches necessary to unravel the complexities of sensory-cognitive interactions. This Research Topic not only advances our understanding of the neural basis of perception and cognition but also lays the groundwork for future studies exploring the dynamic interplay between sensory inputs and higher-order cognitive functions.

Future research should integrate multi-modal and multi-level approaches, spanning single-neuron recordings to large-scale network analyses, and computational modeling to clinical applications. Such efforts are essential to fully elucidate how sensory information is transformed into cognition.

